# Increased Rate of Hospitalization for Diabetes and Residential Proximity of Hazardous Waste Sites

**DOI:** 10.1289/ehp.9223

**Published:** 2006-08-18

**Authors:** Maria Kouznetsova, Xiaoyu Huang, Jing Ma, Lawrence Lessner, David O. Carpenter

**Affiliations:** 1 Department of Epidemiology and Biostatistics, School of Public Health and; 2 Institute for Health and the Environment, University at Albany, Rensselaer, New York, USA

**Keywords:** behavior, diabetes mellitus, dioxins, negative binomial regression, PCBs, persistent pesticides, polychlorinated biphenyls, SES, socioeconomic status, ZIP codes

## Abstract

**Background:**

Epidemiologic studies suggest that there may be an association between environmental exposure to persistent organic pollutants (POPs) and diabetes.

**Objective:**

The aim of this study was to test the hypothesis that residential proximity to POP-contaminated waste sites result in increased rates of hospitalization for diabetes.

**Methods:**

We determined the number of hospitalized patients 25–74 years of age diagnosed with diabetes in New York State exclusive of New York City for the years 1993–2000. Descriptive statistics and negative binomial regression were used to compare diabetes hospitalization rates in individuals who resided in ZIP codes containing or abutting hazardous waste sites containing POPs (“POP” sites); ZIP codes containing hazardous waste sites but with wastes other than POPs (“other” sites); and ZIP codes without any identified hazardous waste sites (“clean” sites).

**Results:**

Compared with the hospitalization rates for diabetes in clean sites, the rate ratios for diabetes discharges for people residing in POP sites and “other” sites, after adjustment for potential confounders were 1.23 [95% confidence interval (CI), 1.15–1.32] and 1.25 (95% CI, 1.16–1.34), respectively. In a subset of POP sites along the Hudson River, where there is higher income, less smoking, better diet, and more exercise, the rate ratio was 1.36 (95% CI, 1.26–1.47) compared to clean sites.

**Conclusions:**

After controlling for major confounders, we found a statistically significant increase in the rate of hospitalization for diabetes among the population residing in the ZIP codes containing toxic waste sites.

Diabetes is one of the leading causes of death and one of the most costly diseases in developed countries. During 1980–2002 the number of people with physician-diagnosed diabetes in the United States increased more than 2-fold, from 5.8 million to 13.3 million. An estimated 5.2 million cases remain undiagnosed. In 2002, total direct and indirect health care costs for people with diabetes amounted to $132 billion [Centers for Disease Control and Prevention (CDC) 2003]. The prevalence of diabetes of all types was 6.3% in the United States in 2002, of which approximately 90–95% of cases is adult-onset, type 2 diabetes (CDC 2003).

Established risk factors for diabetes include age, hyperinsulinemia (a marker for insulin resistance), obesity, genetic factors, and a sedentary lifestyle [Haffner 1998; World Health Organization (WHO) 1994]. Socioeconomic status (SES) is also a risk factor, in that lower income is associated with an increased risk of obesity and sedentary life style (Brancati et al. 1996). The National Health Interview Survey (National Center for Health Statistics 2003) found race, sex, obesity, and age to be effect modifiers for the prevalence of diabetes. Diabetes generally increased more rapidly with obesity among women than among men, but there was no other consistent sex difference. African-American race was a strong risk factor for diabetes, especially among individuals of low SES. After adjustments for racial differences in age, SES, weight, and central adiposity, African Americans remained over twice as likely to have diabetes as whites [odds ratio (OR) = 2.35; 95% confidence interval (CI), 1.49–3.73; *p* = 0.0003] (Brancati et al. 1996).

In addition, recent epidemiologic evidence suggests associations between diabetes and several environmental exposures, including cigarette smoke (Will et al. 2001) and arsenic (Tsai et al. 1999). Dioxin-exposed populations have been found to be at increased risk of diabetes (Bertazzi et al. 1998; Cranmer et al. 2000; Henriksen et al. 1997), and recent studies suggest an association with polychlorinated biphenyl (PCB) exposure (Longnecker and Daniels 2001; Radikova et al. 2004). Some PCB congeners activate the aryl hydrocarbon receptor, and thus are dioxin-like in activity, whereas other congeners have different modes of action (Giesy and Kannan 1998).

Persistent organic pollutants (POPs), such as dioxins, furans, PCBs, and chlorinated pesticides, are complex mixtures of organic molecules that vary in the degree of chlorination. Whereas dioxins and furans are unintended products of incineration and byproducts of some industrial processes, PCBs were manufactured and used primarily as coolants and lubricants in electrical equipment and as hydraulic fluids. The production of PCBs in the United States was discontinued in the late 1970s due to evidence that they, like dioxins and furans, persist in the environment and can cause toxic effects (Agency for Toxic Substances and Disease Registry 2000). The manufacture of most chlorinated pesticides was also stopped in developed countries in the late 1970s or early 1980s. The major routes of exposure to these compounds are ingestion of fish (especially sport fish caught in polluted lakes or rivers), meat and dairy products (Dellinger et al. 1996; Falk et al. 1999), and inhalation of contaminated air near hazardous waste sites (DeCaprio et al. 2005).

The objective of this study was to assess the potential association between residence near hazardous waste sites and hospitalization rates for diabetes among adult residents of New York State (NYS).

## Materials and Methods

### Study population

We used the New York Statewide Planning and Research Cooperative System (SPARCS) to obtain data on diabetes diagnosis among inpatients from 1993–2000. All hospitals regulated by and located in NYS are required to report every diagnosis (up to 15) for each inpatient, upon discharge, to the NYS Department of Health, based on the *International Classification of Disease, 9th Revision, Clinical Modification* (ICD-9-CM; National Center for Health Statistics 1980). The SPARCS data used includes patient age, sex, race, and ZIP code of current residence. New York City (NYC) maintains a separate data set and therefore was not included in this study. The SPARCS data does not identify individuals with multiple hospitalizations or patients in federally regulated hospitals, nor does it include out-of-state health care treatment received by NYS residents. It lists only current, not previous, residences, as previously reported (Sergeev and Carpenter 2005).

There are other important confounders for which information is not contained in the SPARCS dataset. Median household income by ZIP code was obtained from Claritas, Inc. (San Diego, CA) and was used as a proxy for SES. Rates of smoking, consumption of fruits and vegetables, and frequency of exercise (as surrogates for obesity) were obtained for counties (not ZIP codes) along the Hudson River from the Behavioral Risk Factor Surveillance System (BRFSS), as previously reported (Huang et al. 2006).

In this study we examined only two racial groups (Caucasians and African Americans) to reduce variability. These groups comprise 95% of diabetes hospitalizations in NYS exclusive of NYC. We identified all of the hospitalizations that included any of the ICD-9 codes for diabetes mellitus (ICD-9 code 250), which includes all forms of diabetes, and we studied patients 25–74 years of age.

We restricted our regression analysis to the two middle quartile income groups (second and third quartiles), with the median household incomes ranging from $31,107.00 to $51,482.00. Our previous studies have shown that the extremes of SES show different health impacts (Huang et al. 2006; Shcherbatykh et al. 2005). Table 1 shows the characteristics of the study population. Some epidemiologic studies have demonstrated differences in rates of diabetes in urban compared to rural residents (Al-Moosa et al. 2006; Illangasekera et al. 2004; Wild et al. 2004; Zimmet et al. 1983). Therefore, we controlled for population density. Using the Census Bureau classification (U.S. Census Bureau 2002), we considered ZIP codes with > 386 persons per square mile to be urban, and those with ≤ 386 to be rural.

### Assessment of exposure

**Figure 1:**
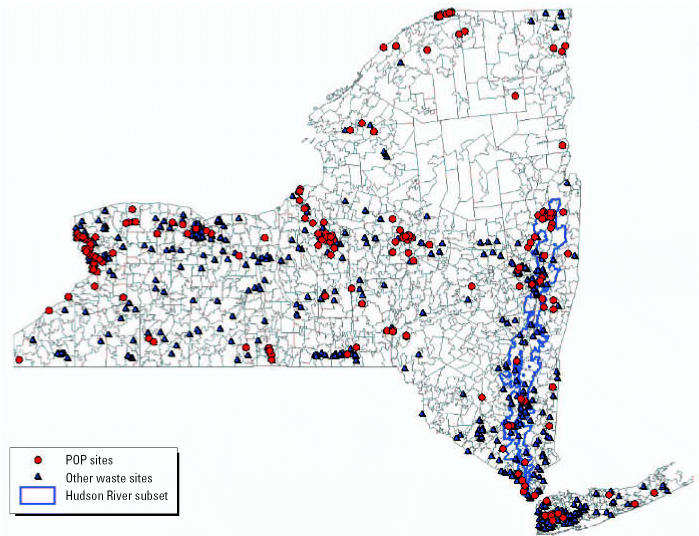
Map of distribution of the waste sites in NYS by ZIP code.

Hazardous waste sites in New York were identified as previously described (Huang et al. 2006; Sergeev and Carpenter 2005; Shcherbatykh et al. 2005). The NYS Department of Environmental Conservation has identified 818 sites (state Superfund sites) that pose a potential threat to human health. The list includes 89 National Priority Sites identified by the U.S. Environmental Protection Agency (EPA 2003). In addition there are six areas of concern, highly contaminated portions of the Great Lakes and St. Lawrence River, in NYS identified by the International Joint Commission (U.S. EPA 2004). We identified the ZIP code(s) in which these sites were located, or in the case of a contaminated body of water, the ZIP code(s) that abuts the site. We classified ZIP codes into three distinct groups. “POP” ZIP codes are 194 ZIP codes that contain or abut one or more hazardous waste sites contaminated with POPs (dioxins/furans, PCBs, persistent pesticides); these include all ZIP codes that abut the six areas of concern and the PCB-contaminated portion of the Hudson River from Hudson Falls to Manhattan (NYC). There were 213 “other” ZIP codes that contain a hazardous waste site containing, for example, volatile organics and metals, but no POPs. The 995 ZIP codes that do not contain or abut any identified hazardous waste site were categorized as “clean” sites. We separately analyzed a subset of the POP sites consisting of the 78 ZIP codes in the PCB-contaminated portion of the Hudson River from Hudson Falls to NYC (30% of all people living in POP-contaminated ZIP codes). Figure 1 shows the location of the waste sites in NYS.

### Statistical analysis

We calculated diabetes hospitalization rates per 100,000 as the number of discharge diagnoses of diabetes divided by the total population residing in the ZIP codes of each category. All statistical analyses were performed with SAS software (version 8.2; SAS Institute Inc., Cary, NC). We modeled the rates of diabetes hospitalization in the different categories of ZIP code as a Poisson process. However, when Poisson regression, a log-linear model, was applied using PROC GENMOD (SAS Institute), the deviance test for the quality of fit of model and the residual plot indicated extra Poisson variation (Woodward 1999). Consequently, we used the negative binomial regression model (Cameron and Trivedi 1998). This model is a log-linear model (i.e., the mean number of discharges is determined by the linear combination of covariates):


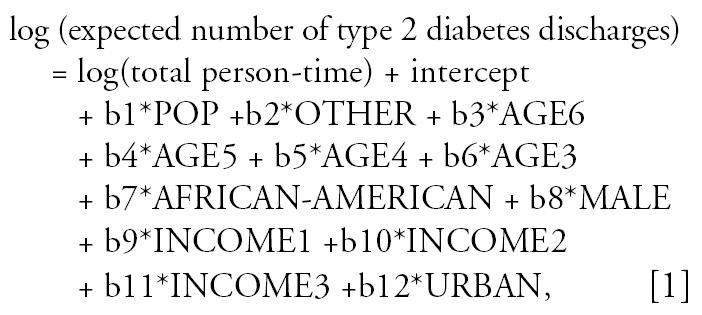


where AGE3 is ages 35–44, AGE4 is 45–54, AGE5 is 55–65, and AGE6 is 65–74; INCOME1 is an annual median household income of $31,107.00–33,708.50, INCOME2 is $33,708.50–37,687.50, and INCOME3 is $37,687.50–42,500.00; URBAN is the ZIP codes with ≥ 386 persons/k^2^; and POP and OTHER are the covariates with the value of zero or 1.

## Results

**Figure 2:**
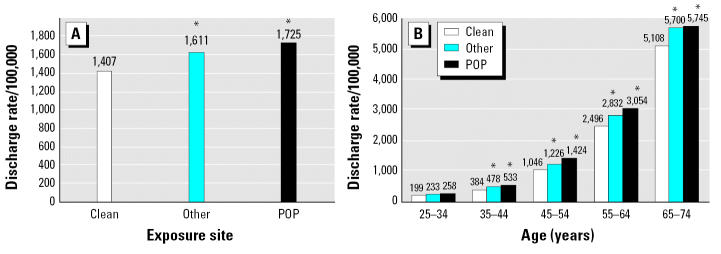
Crude (unadjusted) hospitalization rates for diabetes before modeling for all of the NYS population (except NYC) for ages 25–74 years in clean, “other,” and POP sites (*A*) and broken down by age (*B*). The numbers above the bars indicate the rates per 100,000. *Statistically significant compared to clean sites (*p* < 0.05 ).

Crude analysis showed an increased rate of inpatient hospital diagnosis of diabetes in individuals residing in POP sites compared with “other” waste or clean sites (Figure 2A). The rate of diabetes increased with age and was significantly higher among subjects residing in both POP and other sites compared with clean sites (Figure 2B). The relative increase, especially for POP sites, was greatest at younger ages, suggesting earlier age of onset of diabetes. These data include the full NYS population (except NYC) and not only the two middle quartiles of income that are used below for the regression analysis.

In our analysis of only the two middle quartiles of income (Table 2), after adjusting for the potential confounders (age, race, sex, income, urban/rural population density), the rate ratio (RR) was significantly elevated (23%) among residents of POP sites compared to clean sites. We also found a 25% increase in discharge rates for diabetes in the “other” sites compared with clean sites. The difference between rates in POP sites and “other” sites was not significant. As expected, the overall RRs gradually increased along with age. These results are consistent with previous studies and the national trends (National Center for Health Statistics 2003). African-Americans were 2.6 times more likely to be diagnosed with diabetes than Caucasians. We found no significant difference between the sexes in our sample. As expected, hospitalization rates varied with income, being higher in individuals with lower income. Urban/rural population density was also a significant risk factor, with rates elevated in the urban population (RR = 1.09).

We tested the quality of fit of our negative binomial model. The value of the Pearson chi-square and deviance divided by the number of degrees of freedom was close to 1, which indicates that the fit of the model was adequate.

There are other important confounders for diabetes for which information is not available in the SPARCS dataset, including rates of smoking and obesity and frequency of exercise. We previously reported a comparison of some behavioral factors in counties that abut the contaminated portion of the Hudson River compared with the rest of NYS using data obtained from the BRFSS data set (Huang et al. 2006). The results showed that Hudson River residents got more exercise and ate more fruits and vegetables, both of which are indirect measures of the incidence of obesity. The current smoking rates along the Hudson River are less than in the rest of the state. The residents of the ZIP codes along the contaminated portion of the Hudson River have higher average incomes (Huang et al. 2006); there are fewer families with incomes < $24,999, and more families with incomes > $50,000. The important conclusion is that Hudson River residents have higher incomes, live a healthier lifestyle, and smoke less than other New Yorkers.

We determined the rates of diabetes diagnosis among hospitalized residents in the 78 ZIP codes along the PCB-contaminated portion of the Hudson River compared with the “other” (non-POP) sites and the clean sites. The results of the negative binomial model for this population are shown in Table 3. The rates of diabetes diagnosis were 36% higher among Hudson River residents than those of clean sites, in spite of the fact that they have a healthier lifestyle.

## Discussion

Diabetes is not one of the diseases usually thought to be secondary to environmental contaminants. However, although chemical contaminants are certainly not the only, or perhaps even the major, risk factor for diabetes, they are a factor that must be considered. In a study of U.S. Air Force personnel who dropped Agent Orange in Vietnam, Henriksen et al. (1997) found a highly significant relationship between exposure to dioxin and onset and severity of diabetes in individuals with the highest exposure. This resulted in a committee of the Institute of Medicine (2001) concluding that there was suggestive evidence of an association between dioxin exposure and diabetes. Pesatori et al. (1998) and Bertazzi et al. (1998) found elevated rates of diabetes in individuals exposed to dioxin in Seveso, Italy, after an explosion of a pesticide plant in which dioxin was an unwanted by-product. Vena et al. (1998) reached a similar conclusion in a WHO study of workers exposed to dioxins during production of phenoxyacid herbicides and chlorophenol. Cranmer et al. (2000) studied individuals exposed to dioxin from the site of a former pesticide manufacturing plant in Arkansas; they found that plasma insulin concentrations were significantly higher in individuals with elevated dioxin levels, and they concluded that elevated serum dioxin levels cause insulin resistance.

Longnecker et al. (2001) studied 2,245 pregnant women, 44 of whom had diabetes. The mean serum PCB level in the women with diabetes (3.77 ppb) was 30% higher than that in the controls (2.79 ppb), and the relationship of PCB level to adjusted OR for diabetes was linear. Taking PCB levels < 2.50 ppb to have an OR of 1.0, the OR was 2.9 for PCB levels of 2.50–3.75 ppb, 4.4 for PCB levels of 3.75–5.00 ppb, and 5.1 for PCB levels of > 5.0 ppb. All values were statistically significant. Thus, this study shows a dose–response relationship. In a population-based study, Fierens et al. (2003) found, after adjustment for age and other covariates, that total toxic equivalence and concentrations of the sum of 12 marker PCBs were 62% and 39% higher, respectively, than in controls. The ORs were 5.1 (95% CI, 1.18–21.7) for dioxins, 13.3 (95% CI, 3.31–53.2) for coplanar PCBs, and 7.6 (95% CI, 1.58–36.3) for 12 marker PCBs in the upper decile of exposure. Radikova et al. (2004) reported an elevated incidence of impaired fasting glucose and incidence of diabetes in an exposed human population with serum measurements of PCBs and chlorinated pesticides.

Animal studies also show that PCB and dioxin exposure increases risk of diabetes. Nishizume et al. (1995) showed that rats given Kanechlor 400 showed depressed insulin sensitivity, which increased with the duration of PCB exposure; Kanechlor 400 also disturbed glucose and lipid metabolism and elevated serum lipids. Stahl (1995) reported that dioxin alters enzyme activity related to glucose metabolism in rat liver cells. Others have demonstrated morphologic changes of the beta cells in the pancreas after PCB exposure (Kimbrough et al. 1972; Wassermann et al. 1975). Boll et al. (1998) demonstrated that gluconeogenic enzymes in rat liver are altered after PCB exposure.

Although we are not aware of previous studies on diabetes in relation to site of residence, others have reported elevated disease in individuals living near hazardous waste sites, including rates of congenital anomalies (Dolk et al. 1998; Geschwind et al. 1992; Malik et al. 2004), low birth weight (Elliott et al. 2001), and end-stage renal disease (Hall et al. 1996). Gaffney et al. (2005) demonstrated that human serum levels of dieldrin, one of the chlorinated pesticides, decreased significantly in an inverse relation to residential distance from a contaminated site. However others (Pless-Mulloli et al. 2005) have not demonstrated any elevation in serum levels of dioxins or PCBs among individuals living near a chemical complex.

Results of the present study demonstrate a statistically significant increase in the rate of hospitalization for diabetes after controlling for major potential confounders among the adult population residing in the ZIP codes containing toxic waste sites, particularly waste sites containing POPs. However, our results do not constitute proof of cause and effect for a variety of reasons. Residence near a hazardous waste site was our only measure of exposure, and it is a very crude measure. We are aware of the methodical limitations in this study. The exposure and response are measured only at an aggregated level rather than for individuals, which introduces a possible aggregation bias. Although there are several individual risk factors for diabetes that we did not control for (e.g., diet, exercise, and smoking), they are only confounders when their frequency in the subpopulation is associated with exposure.

We do not have personal behavioral information on individuals, and there are many known risk factors for diabetes. The BRFSS data from the counties near the contaminated portion of the Hudson River indicate that, on average, individuals living there get more exercise and eat more fruits and vegetables (a surrogate measure of obesity) than other residents; but again, these are aggregated data and may not apply to the specific individuals with diabetes. The same applies to the SES data, which are based on ZIP code; the data represent the average family income in that ZIP code, and not information on the patients diagnosed with diabetes. We have no information on duration of residence in the current ZIP code, which could lead to a migration bias that can affect the validity of ecologic studies, particularly for long-latency, chronic diseases (Tong 2000). We have no control for past occupational or residential exposures that are not correlated with an existing and identified hazardous waste site. Ashton et al. (1999) demonstrated geographic variation in utilization of Veterans Affairs hospitals. This is not a factor for which we have control in this study; also, because our population consists of inpatients, this is a potential source of bias and measurement error. However, Twigger and Jessop (2000) did not find any relationship between travel time to a hospital and rates of admission for diabetes.

Despite the limitations, one might argue that if we find such clear elevations in rates of diabetes when our exposure assessment is so crude, the real relationship between disease and exposure is likely much stronger. Our observations suggest that residence near a hazardous waste site constituted a risk of exposure to these individuals at some time in the past, and this has led to an increased risk of developing diabetes. The risk may still exist. The most likely pathway of exposure is air transport of contaminants; contaminated particulates may be ingested, and both vapor-phase and particulate-bound contaminants may be inhaled. It is unlikely that there are different ingestion patterns of contaminated fish or other food products within specific ZIP codes of residence. Although our observations must be viewed as being hypothesis generating, they provide additional support for a relationship between exposure to environmental contaminants, especially POPs, and risk of diabetes. Further study is necessary to determine whether this is a causative relationship; if so, we need to determine the relative contribution of POPs.

## Correction

In the original manuscript published online, RRs and 95% CIs in the Abstract, all values in Table 2 and 3, and values in the text referring to these tables were underestimated. Also, the numbers and percentages for the the clean group were incorrect in Table 1. All of these have been corrected here.

## Figures and Tables

**Table 1:** Distribution of characteristics in the study population.

Characteristic	Diabetic subjects No. (%)	Total person-years^a^ No. (%)
Exposure	125,283 (37.3)	8,966,252 (41.6)
Total POPs	119,821 (35.7)	7,112,176 (33.0)
Hudson POPs	54,942 (45.9)^b^	2,871,808 (40.4)^b^
Other	90,448 (27.0)	5,491,372 (25.5)
Clean	125,283 (37.3)	8,966,252 (41.6)
Age (years)		
65–74	151,701 (45.2)	2,771,652 (12.8)
55–64	91,605 (27.3)	3,194,612 (14.8)
45–54	54,784 (16.3)	4,485,828 (20.8)
35–44	25,559 (7.6)	5,778,532 (26.8)
25–34	11,903 (3.5)	5,339,176 (24.8)
Race		
African American	41,543 (12.4)	1,487,372 (6.9)
Caucasian	294,009 (87.6)	20,082,428 (93.1)
Sex		
Male	164,909 (49.1)	10,517,272 (48.8)
Female	170,643 (50.9)	11,052,528 (51.2)
Income (US$)		
31,107.00–33,708.50	78,334 (23.3)	4,340,736 (20.1)
33,708.50–37,687.50	82,939 (24.7)	4,807,208 (22.3)
37,687.50–42,500.00	85,159 (25.4)	5,662,812 (26.3)
42,500.00–51,482.00 (reference)	89,120 (26.6)	6,759,044 (31.3)

a Sum of the population by ZIP code, 1993–2000.

b Percentage of total POPs.

**Table 2:** Results of regression analysis for diabetes discharges (POP sites).

	Coefficient	SE	RR (95% CI)	*p*-Value
Site				
POP	0.208	1.04	1.23 (1.15–1.32)	< 0.0001
Other	0.222	1.04	1.25 (1.16–1.34)	< 0.0001
Clean (reference)	0.000	1.00	1.00	
Age (years)				
65–74	3.151	1.05	23.36 (21.29–25.63)	< 0.0001
55–64	2.596	1.05	13.41 (12.22–14.71)	< 0.0001
45–54	1.774	1.05	5.89 (5.37–6.47)	< 0.0001
35–44	0.769	1.05	2.16 (1.96–2.37)	< 0.0001
25–34 (ref)	0.000	1.00	1.00	
Race				
African American	0.953	1.03	2.59 (2.45–2.75)	< 0.0001
Caucasian (reference)	0.000	1.00	1.00	
Sex				
Male	–0.023	1.03	0.98 (0.92–1.04)	< 0.4445
Female	0.000	1.00	1.00	
Income (US$)				
31,107.00–33,708.50	0.318	1.04	1.37 (1.27–1.49)	< 0.0001
33,708.50–37,687.50	0.308	1.04	1.36 (1.25–1.48)	< 0.0001
37,687.50–42,500.00	0.068	1.04	1.06 (0.99–1.16)	0.1033
42,500.00–51,482.00 (reference)	0.000	1.00	1.00	
Urban	0.090	1.03	1.09 (1.03–1.16)	0.0023
Rural (reference)	0.000	1.00	1.00	

**Table 3:** Results of regression analysis for diabetes discharges (Hudson River POP subset).

	Coefficient	SE	RR (95% CI)	*p*-Value
Site				
Hudson River POP subset	0.311	1.04	1.36 (1.26–1.47)	< 0.0001
Other	0.222	1.04	1.25 (1.16–1.35)	< 0.0001
Clean (reference)	0.000	1.00	1.00	
Age (years)				
65–74	3.158	1.05	23.53 (21.27–26.03)	< 0.0001
55–64	2.618	1.05	13.71 (12.39–15.17)	< 0.0001
45–54	1.787	1.05	5.97 (5.39–6.61)	< 0.0001
35–44	0.762	1.05	2.14 (1.93–2.38)	< 0.0001
25–34 (reference)	0.000	1.00	1.00	
Race				
African American	0.954	1.03	2.60 (2.44–2.77)	< 0.0001
Caucasian (reference)	0.000	1.00	1.00	
Sex				
Male	0.007	1.03	1.01 (0.95–1.07)	0.8271
Female	0.000	1.00	1.00	
Income (US$)				
31,107.00–33,708.50	0.332	1.05	1.39 (1.28–1.52)	< 0.0001
33,708.50–37,687.50	0.311	1.05	1.36 (1.25–1.49)	< 0.0001
37,687.50–42,500.00	0.082	1.05	1.08 (0.99–1.18)	0.0700
42,500.00–51,482.00 (reference)	0.000	1.00	1.00	
Urban	0.108	1.03	1.11 (1.04–1.19)	0.0008
Rural (reference)	0.000	1.00	1.00	
